# Supplementary material for: Causal Relationship between Aging and Anorexia Nervosa: A White-Matter-Microstructure-Mediated Mendelian Randomization Analysis
Source: Biomedicines. 2024 Aug 16;12(8):1874. doi: 10.3390/biomedicines12081874 (PMC11351342; doi:10.3390/biomedicines12081874)
Supplement: Supplementary file 1 [file biomedicines-12-01874-s001.zip › Supplementary Figure/Figure S4/Singe SNP plot of MR analysis for TL to ubm-b-1797.pdf]

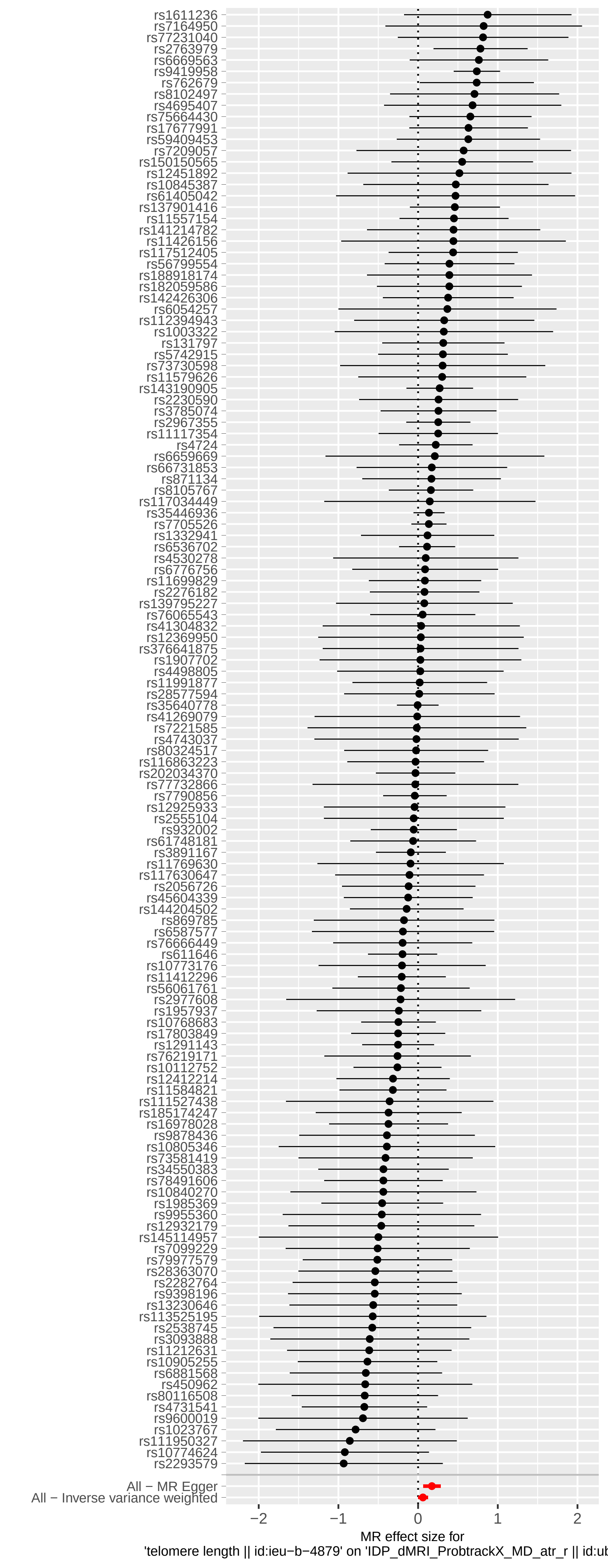
rs1611236
rs7164950
rs77231040
rs2763979
rs6669563
rs9419958
rs762679
rs8102497
rs4695407
rs75664430
rs17677991
rs59409453
rs7209057
rs150150565
rs12451892
rs10845387
rs61405042
rs137901416
rs11557154
rs141214782
rs11426156
rs117512405
rs56799554
rs188918174
rs182059586
rs142426306
rs6054257
rs112394943
rs1003322
rs131797
rs5742915
rs73730598
rs11579626
rs143190905
rs2230590
rs3785074
rs2967355
rs11117354
rs4724
rs6659669
rs66731853
rs871134
rs8105767
rs117034449
rs35446936
rs7705526
rs1332941
rs6536702
rs4530278
rs6776756
rs11699829
rs2276182
rs139795227
rs76065543
rs41304832
rs12369950
rs376641875
rs1907702
rs4498805
rs11991877
rs28577594
rs35640778
rs41269079
rs7221585
rs4743037
rs80324517
rs116863223
rs202034370
rs77732866
rs7790856
rs12925933
rs2555104
rs932002
rs61748181
rs3891167
rs11769630
rs117630647
rs2056726
rs45604339
rs144204502
rs869785
rs6587577
rs76666449
rs611646
rs10773176
rs11412296
rs56061761
rs2977608
rs1957937
rs10768683
rs17803849
rs1291143
rs76219171
rs10112752
rs12412214
rs11584821
rs111527438
rs185174247
rs16978028
rs9878436
rs10805346
rs73581419
rs34550383
rs78491606
rs10840270
rs1985369
rs9955360
rs12932179
rs145114957
rs7099229
rs79977579
rs28363070
rs2282764
rs9398196
rs13230646
rs113525195
rs2538745
rs3093888
rs11212631
rs10905255
rs6881568
rs450962
rs80116508
rs4731541
rs9600019
rs1023767
rs111950327
rs10774624
rs2293579
All − MR Egger
All − Inverse variance weighted
−2
−1
0
1
2
MR effect size for
'telomere length || id:ieu−b−4879' on 'IDP_dMRI_ProbtrackX_MD_atr_r || id:ub
